# Testing the Efficacy of the Red-Light Purple-Light Games in Preprimary Classrooms in Kenya

**DOI:** 10.3389/fpsyg.2021.633049

**Published:** 2021-03-11

**Authors:** Michael T. Willoughby, Benjamin Piper, Katherine Merseth King, Tabitha Nduku, Catherine Henny, Sarah Zimmermann

**Affiliations:** ^1^Education and Workforce Development, RTI International, Research Triangle Park, NC, United States; ^2^International Education, RTI International, Nairobi, Kenya; ^3^International Education, RTI International, Washington, DC, United States; ^4^International Education, RTI International, Research Triangle Park, NC, United States; ^5^Research Computing Division, RTI International, Research Triangle Park, NC, United States

**Keywords:** intervention, executive function, low and middle income countries, Global South, early childhood

## Abstract

This study adapted and tested the efficacy of the Red-Light Purple-Light (RLPL) games for improving executive function (EF) skills in preprimary classrooms in Nairobi, Kenya. A cluster randomized controlled trial was used to evaluate the efficacy of the adapted RLPL intervention. Specifically, 24 centers (including 48 classrooms) were randomized to the RLPL or a wait-list control condition. Consistent with previous studies, participating classrooms delivered 16 lessons across an 8-week intervention period. A total of 479 children were recruited into the study. After exclusions based on child age and data quality, 451 and 404 children (90% retention) had completed computerized assessments of EF skills at pre- and posttest assessments, respectively. Children in the RLPL centers did not demonstrate any improvements in EF skills relative to their peers in the wait-list control condition (Cohen’s *ds* = −0.14 to 0.03, all *p*s > 0.20). Exploratory tests of moderators (language of assessment, grade, school type, baseline ability) were also all null. Results are discussed with respect to measurement limitations and contextual factors that may explain the null results of RLPL on EF skills in young children in Kenya.

## Highlights

- A cluster randomized controlled trial was used to test the efficacy of the Red-Light Purple-Light (RLPL) classroom games in Nairobi County, Kenya.
- In contrast to studies in the United States and New Zealand, children who were exposed to RLPL games did not demonstrate improvements in executive function skills.
- Exploratory tests of moderators (language of assessment, grade, school type, and baseline ability) were also all null.
- This study raises questions about the transportability of efficacious preschool interventions from high- to low- and middle-income country contexts.

## Introduction

Executive function (EF) skills are cognitive processes that support goal-directed behavior and that facilitate the transition to formal schooling (Blair and Raver, 2015). EF skills can be improved through intervention, and early childhood may be a sensitive period for interventions. Although most of what is known about EF skills is derived from research in high-income countries, EF skills are germane to early learning and school readiness in low- and middle-income countries (LMICs) (Obradović and Willoughby, 2019). There is global interest among educators and policymakers in identifying practical and developmentally appropriate strategies for improving children’s EF skills.

Efforts to improve children’s EF skills have taken multiple forms, including cognitive training programs (Aksayli et al., 2019), physical activity programs (Erickson et al., 2019), classroom curricula that specifically target EF (Solomon et al., 2017), and the promotion of classroom management procedures and high-quality instructional practices (Bierman et al., 2008; Raver et al., 2011). Takacs and Kassai (2019) conducted a series of meta-analyses, involving nearly 9,000 children from 90 studies, of EF interventions that involved children aged 2–12 years. Whereas explicit cognitive training and self-regulatory programs were associated with moderate-sized increases in EF skills, physical activity and EF-specific classroom curricula were associated with small increases in EF skills. Some programs were differentially effective for typically developing compared to atypically developing children.

In addition to evidence that EF skills are malleable, interventions may be especially effective if delivered in early childhood. Johnston et al. (2009) postulated that neural networks that are relatively unspecialized and undifferentiated in early development become increasingly specialized over time through normative experience. Early interventions may have broader impact on developing neural networks than can interventions later in development. A review of cognitive training programs provided some empirical support for this speculation. Wass et al. (2012) reported that cognitive interventions were associated with a more widespread transfer of effects across cognitive domains when delivered earlier in development (*r* = −0.47).

Most of what we know about EF interventions comes from high-income countries. Most studies that were included in the Takacs and Kassai (2019) meta-analysis were delivered in the United States and Western Europe. However, evidence supporting the criterion validity, biological basis, and family correlates of EF skills in early childhood is comparable across LMIC contexts (Obradović and Willoughby, 2019). We are unaware of any intervention studies in LMICs that specifically targeted improvements in EF skills in early childhood. Rather, most studies have focused on broader targets—for example, maternal sensitivity and/or nutrition—and considered EF skills as one of many outcomes (e.g., Yousafzai et al., 2016; Boivin et al., 2017; Roberts et al., 2020). Here, we describe efforts to identify and test an EF-focused intervention delivered in preprimary classrooms in Kenya.

Kenyan law establishes that every child has the right to free and compulsory early childhood education (Republic of Kenya, 2005). However, access, equity, and the quality of education in the preprimary level is constrained by an inadequate number of preprimary centers, undertrained teachers, and limited teaching and learning materials (Piper et al., 2018). Some preprimary programs have been effective in increasing school readiness in Kenya. For example, the Tayari program, which was delivered in over 2,500 preprimary centers in Kenya, increased preacademic and social-emotional outcomes using structured interventions through government personnel and training systems (APHRC, 2019). Here, we tested whether an intervention that specifically targeted EF skills could be delivered within preprimary classrooms that had previously participated in the Tayari intervention.

Three pragmatic issues informed our selection of an EF intervention. First, preprimary class sizes in Kenya are large (Abuom et al., 2018), which makes “pull-out” style interventions impractical. Second, preprimary classes in Kenya have relatively limited teaching and learning materials (World Bank, 2015). Third, teachers are typically provided limited training after their preservice preparation programs. We identified the Red-Light Purple-Light (RLPL) circle-time games intervention as a promising candidate for use in Kenya (Tominey and McClelland, 2011). RLPL can be delivered in a classroom-wide arrangement, requires minimal materials, and does not require intensive training for teachers to implement successfully. RLPL attempts to improve preschool children’s behavioral regulation skills, which are closely related to and correlated with EF skills (McClelland et al., 2014), using group-based music and movement games. The RLPL intervention involves 16 group-based activities (each 20–30 min) delivered in preschool classrooms across 8 weeks.

Five previous studies have documented the efficacy of the RLPL intervention in improving children’s behavioral regulation and EF skills. Although RLPL was initially evaluated using a small-group, pull-out format (Tominey and McClelland, 2011), four subsequent studies that involved classroom-wide delivery of RLPL activities documented moderate-sized improvements in behavioral regulation and EF skills, with Cohen’s *d*s = 0.31–0.38 (Schmitt et al., 2015; Duncan et al., 2018; McClelland et al., 2019; Keown et al., 2020). Given the ease of use and replicated evidence of efficacy in the United States and New Zealand, we adapted and tested the RLPL for use in Kenya.

## Method

### Participants and Procedures

This study evaluated the efficacy of the RLPL intervention in 48 classrooms in 24 preprimary centers in Nairobi County, Kenya. Given funding constraints, we limited centers to those that had previously participated in the Tayari program (Ngware et al., 2016). We identified six zones of schools that successfully implemented Tayari and that were accessible to the Tayari Nairobi office. We randomly selected one public and one low-cost private school zone for inclusion in this study. We randomized half of the centers within these zones to RLPL or wait-list control conditions. The treatment group included ten public centers overseen by the Kenyan Ministry of Education and 14 centers owned and run by private individuals [i.e., Alternative Provision of Basic Education and Training (APBET) centers]. Teachers in the centers that were assigned to the wait-list control condition received RLPL training and materials after the active study period was complete. Random assignment at the center level helped to mitigate potential threats of contamination. This study was approved by the National Commission for Science, Technology, and Innovation and by the Kenya Medical Research Institute.

Two classrooms (preprimary 1: 4-year-olds; preprimary 2: 5-year-olds) per center were selected to participate. All the children in the two classrooms participated in the intervention, and ten children (balanced by gender) per classroom were randomly selected to participate in data collection, with 100% of sampled children’s parents providing written consent. The high consent rate was achieved due to the positive relationships that were built from the Tayari intervention, as well as the diligence of teacher follow-up with parents. Children completed the same EF assessments in the week before and after the 8-week RLPL intervention period. Many children in urban Nairobi are exposed to both English and Kiswahili, and the Tayari intervention explicitly had activities in both languages. Although English is the formal language of instruction in Kenya, many children in Nairobi are more adept at Kiswahili. Consistent with our previous work (Willoughby et al., 2019a), assessors determined the language of assessment during a rapport-building conversation with each child.

We collected data from 479 students at pretest and 438 students at posttest. However, we excluded children who were 7 years or older at the pretest assessment or for whom there were questions about data quality (e.g., a few children appeared to have two assessments conducted at pre- or posttest). Assessors also occasionally switched the language of instruction during an assessment occasion because of concerns about children’s task comprehension. In rare instances, assessors made different decisions about language across pretest and posttest. To improve data quality, we also excluded children who performed EF tasks in more than one language so that EF task performance was not confounded by children’s listening comprehension skills. After all age and data quality exclusions, we had usable data for 451 students at pretest (50% male; *M* = 4.8, SD = 0.8, range = 3–6 years old; 61% APBET schools; 61% assessments in Kiswahili) and 404 students at posttest (50% male; *M* = 4.7, SD = 0.7, range = 3–6 years old; 63% APBET schools; 61% assessments in Kiswahili).

### Teacher Training and Support

The intervention team undertook a review and revision of the RLPL teacher training materials (see Supplementary Material). A 3-day group training was provided to 21 teachers whose schools were randomly assigned to receive the RLPL condition. Three teachers who were unable to attend the group training were trained individually. Unfortunately, these teachers subsequently left their positions. Some of these classrooms were merged with others in the same center, but at least one classroom was led by temporary teachers who did not participate in training. The training protocol largely corresponded to the activities from the RLPL developer. Teachers were provided with the adapted teacher manual and materials and provided with multiple opportunities to model and practice activities. During the implementation period, the project staff who led teacher training completed classroom observations to monitor RLPL implementation and provided teachers with coaching when necessary, although few of the teachers were observed implementing the last (and more complex) RLPL activities.

**TABLE 1:** Item frequencies for fidelity observations.

Item	Percent,%	
	No	Yes
Welcome song sang	0.0	100.0
Goodbye song sang	2.9	97.1
Teacher clearly explained rules	22.9	77.1
Teacher checked for student understanding	61.4	38.6
Teacher modeled appropriate actions	1.4	98.6
Teacher had appropriate materials	0.0	94.3
Teacher used appropriate materials	0.0	92.9
Teacher selected child(ren) to lead activity	10.0	80.0
Length of session was appropriate	14.3	85.7
Teacher seemed prepared	11.4	88.6
Was the environment conducive	42.8	57.1

*Frequencies are summarized across all 70 observations. Some items do not sum to 100% because of structurally missing data (i.e., some items were not applicable at the time of a fidelity observation due to the nature of the activity being observed).*

### Measures

Given time and cost constraints, our evaluation of the RLPL program was limited to student performance on tablet-based EF skills assessments that were previously validated for use in Kenya [see Willoughby et al. (2019a) for a full description of task development]. The same tasks were administered at pre- and posttest assessments in the days immediately before and after the delivery of the RLPL intervention. All assessments occurred in preprimary centers. Assessors began with a simple warm-up task that acclimated children to using the touch screen. Children also completed a simple reaction-time task and five EF tasks (described below). All the EF tasks followed a similar structure that involved the assessor reading a fully standardized script including task instructions, demonstrating how to complete sample items, and then presenting training items to the child. Tasks were automatically discontinued if the child was unable to independently pass the training items after two attempts. Information regarding the internal consistency and quality ratings for each task (reported below) are derived from the pretest.

#### Spatial Conflict Arrows

This 36-item spatial conflict task measured inhibitory control (IC) and cognitive flexibility. In this task, children were instructed to touch a button (on the right or the left side of the screen) to which an arrow was pointing. First the arrows appeared above the button to which they were pointing (congruent position), next the arrows appeared above the opposite button (incongruent position), and finally they appeared in a combination of previous locations (mixed condition). Mean accuracy for the number of incongruent items (from incongruent and mixed conditions) was used to represent performance (α = 0.95). The mean quality rating was 2.3 (SD = 0.60; 7% low, 56% acceptable, 37% high quality).

#### Silly Sounds Stroop

This 17-item Stroop-like task measured IC. Each item presented pictures of a dog and a cat and the sound of either a dog barking or a cat meowing. The child was instructed to touch the picture of the animal that did not make the sound (e.g., touching the cat when hearing a dog bark). Mean accuracy across all items was used to represent performance (α = 0.91). The mean quality rating was 2.5 (SD = 0.60; 6% low, 38% acceptable, 56% high quality).

#### Animal Go/No-Go

This 40-item go/no-go task measured IC. Individual pictures of animals were presented, and children were instructed to touch a centrally located button on their screen every time that they saw an animal, except when that animal was a pig. Item responses that were faster than 400 ms were considered too fast to be plausible and were set to missing. If an item was omitted, children were given an accuracy score of zero, and reaction time was not recorded. Mean accuracy across the eight no-go items was used to represent performance (α = 0.81). The mean quality rating was 2.5 (SD = 0.60; 6% low, 37% acceptable, 57% high quality).

#### Something’s the Same

This 19-item task measured attention shifting. For each item, children were tasked with categorizing pictures based on one dimension (e.g., color) and then categorizing them by another dimension (e.g., shape). The mean accuracy of responses was used to represent task performance, despite a low level of internal consistency (α = 0.50). The mean quality rating was 2.3 (SD = 0.60; 7% low, 58% acceptable, and 34% high quality).

#### Pick the Picture

This 32-item (10 sets of pictures) task measured working memory. Children were presented with sets of pictures that ranged from two to six items. Children initially were instructed to touch any picture of their choice. On subsequent trials within that set, the pictures were presented in different locations, and children were instructed to pick a picture that had not yet been touched. Mean performance across the ten picture sets was used to represent task performance (α = 0.75). The mean quality rating was 2.3 (5% low, 56% acceptable, and 39% high quality).

We previously documented the benefits of combining individual task scores into composites to improve reliability (Willoughby et al., 2016, 2017). We computed an overall EF composite based on mean performance across five EF tasks. We also computed an IC composite (mean performance across three tasks) because previous evaluations of RLPL primarily relied on the Head Toes Knees Shoulder (HTKS) task, which requires IC.

### Analysis Plan

Mixed linear models were used to test all study hypotheses. Unconditional mixed linear models were used to characterize the hierarchical data structure. Conditional mixed linear models were estimated in which each posttest score was regressed on the corresponding pretest score, pretest simple reaction time, and treatment condition. Pretest scores were included as a covariate to improve the statistical power to detect treatment effects (Raudenbush et al., 2007). Simple reaction time was included as a covariate because it is distinct from but contributes to EF performance (Willoughby et al., 2018). Conditional models were re-estimated four times to test for potential moderators of treatment, including school type (APBET vs. public), grade (preprimary 1 vs. 2), language of EF task administration (Kiswahili vs. English), and children’s pretest performance (continuous scores). To be clear, tests of moderation were exploratory (hypothesis generating) and were not motivated by *a priori* expectations for differential impacts of RLPL on EF outcomes.

## Results

### Descriptive Statistics

#### Fidelity of Implementation

Two individuals who led teacher training completed a total of 70 fidelity observations of classrooms when they were implementing RLPL activities. The median number of observations conducted was three per classroom (range = 1–10). Variations in the number of completed observations primarily reflected differences in teachers’ willingness to be supported. Variation in teacher willingness to receive support is typical in Kenya and was also encountered in the Tayari program. Given their voluntary role in this study, we respected teacher wishes about whether they were observed. Fidelity observations involved the completion of a project-developed checklist that included multiple indicators of implementation quality. The results of each observation were reviewed with teachers to improve the fidelity of implementation. Frequencies for items on the fidelity checklist are summarized in Table 1. Except for two items (“*Teacher checked for student understanding*,” which was endorsed in 38% of observations, and “*Was the environment conducive for the children*,” which was endorsed in 57% of observations), the rates of item endorsement were uniformly high (77%–100% endorsement). Observers also rated the overall lesson implementation quality using a subjective, 3-point Likert scale. Across all observations, 26, 71, and 2% were deemed “very good,” “good,” and “poor,” respectively. A synopsis of the number of fidelity observations, the average number of students, and the proportion of fidelity items that were endorsed in each treatment classroom is summarized in Table 2. Notably, classes varied appreciably in size (median = 30; range = 8–68 students). The smaller classrooms reflect wide ranges of classroom size rather than low attendance. Overall, teachers were observed to complete most of the indicators of implementation quality (median = 86%). In sum, the RLPL intervention was delivered as intended in most participating classrooms, although we had less data on the fidelity of implementation of the last few activities.

**TABLE 2:** Classroom level information from fidelity observations.

Center	Preprimary grade	Total observations	Students present	Fidelity score
	(1, 2)	Count	*M* (SD)	*M* (SD)
1	1	1	11 (–)	1.00 (–)
	2	1	8 (–)	0.64 (–)
2	2	2	27 (1)	0.91 (0.13)
3	1	6	43 (5)	0.85 (0.07)
	2	7	50 (8)	0.77 (0.14)
4	1	6	32 (1)	0.94 (0.07)
	2	10	37 (5)	0.85 (0.10)
5	1	3	68 (25)	0.84 (0.07)
	2	5	35 (5)	0.91 (0.11)
6	1	1	9 (–)	0.91 (–)
	2	1	8 (–)	0.91 (–)
7	1	2	28 (3)	0.79 (0.17)
8	1	3	32 (3)	0.79 (0.19)
	2	3	46 (5)	0.77 (0.24)
9	1	1	30 (–)	1.00 (–)
10	1	3	26 (4)	0.85 (0.19)
	2	3	20 (0)	0.88 (0.14)
11	1	3	39 (1)	0.91 (0.00)
	2	2	31 (0)	0.80 (0.03)
12	1	3	23 (2)	0.76 (0.10)
	2	4	26 (1)	0.63 (0.21)

*Fidelity observations were only completed in 21 of the 24 classrooms that were assigned to the RLPL condition. Three classrooms had to be merged within existing classrooms following unexpected teacher turnover. The fidelity score is the mean of 11 fidelity items that were listed in Table 1.*

#### Executive Function Composites

A synopsis of EF task completion and performance appears in Table 3. At each visit, between 97 and 99% of children attempted each of the five EF tasks. Among children who attempted a task, 75%–99% of children successfully completed training items and proceeded with test items (i.e., each EF task was automatically discontinued if a child was unable to pass training items). Children completed an average of 4.4 (SD = 0.9) of the five EF tasks at pretest (all children completed at least one task, and 85% of children completed four or five tasks). Similar rates of task completion were evident at the posttest assessment (*M* = 4.5 tasks; 88% of children completed four or five tasks).

**TABLE 3:** Completion rates and descriptive statistics for individual executive function tasks.

Task	Pretest						Posttest					
	% Passed			Accuracy			% Passed			Accuracy		
	Total	SWA	ENG	*N*	*M* (SD)	% Ceiling	Total	SWA	ENG	*N*	*M* (SD)	% Ceiling
ARR	76	68	88	341	0.58 (0.38)	21	73	73	74	296	0.64 (0.38)	29
SSS	93	93	93	418	0.82 (0.22)	30	97	97	97	391	0.87 (0.18)	40
AGNG	84	80	91	379	0.74 (0.31)	38	92	93	90	370	0.84 (0.24)	49
STS	87	85	92	394	0.64 (0.14)	1	88	88	88	355	0.67 (0.16)	4
PTP	98	99	97	442	0.68 (0.12)	0	99	98	99	397	0.70 (0.11)	0

*Pretest total *N* = 451; posttest total *N* = 404; SWA, Swahili; ENG, English; M, mean; SD, standard deviation; ARR, spatial conflict arrows (inhibitory control); SSS, silly sounds stroop (inhibitory control); AGNG, animal go/no-go (inhibitory control); STS, something the same (attention shifting); PTP, pick the picture (working memory). % Passed refers to the proportion of children who successfully completed training items (a precondition for the test items to be administered). Ceiling refers to the proportion of students who completed a task and who answered 100% of the test items correctly.*

At pretest, children who had tasks administered in English were more likely to complete the Arrows (88% vs. 68%, *p* < 0.0001) and Animal Go/No-Go (91% vs. 80%, *p* = 0.003) tasks than were children whose tasks were administered in Kiswahili. No differences in the rates of individual task completion as a function of the language of administration were evident at posttest (all *p*s > 0.05; see Table 3). Moreover, the total number of completed tasks did not differ as a function of the language as of administration at pretest (*M*s = 4.5 vs. 4.2 for English and Kiswahili, respectively, *p* = 0.08) or *t* posttest (*M*s = 4.5 and 4.5 for English and Kiswahili, respectively, *p* = 0.86).

Children performed especially well on IC tasks (see Table 3). At pretest, ceiling effects (i.e., answering 100% of the test items correctly) were evident for 21, 30, and 38% of the students who completed the Arrows, Silly Sounds Stroop, and Animal Go/No-Go tasks, respectively. At posttest, ceiling effects increased to 29, 40, and 49% for these three tasks. In comparison, ceiling effects were negligible for Something’s the Same and Pick the Picture tasks (0–4% across pre- and posttest assessments).

Executive function tasks were modestly positively correlated with each other at both pretest (*r*s = 0.12–0.29) and posttest (*r*s = 0.10–0.29) assessments (see Table 4). Consistent with our earlier work (Willoughby et al., 2016), a principal components analysis of the pretest data indicated that the first eigenvalue explained 39% of the variation among EF task correlations, with the remaining eigenvalues <1. A similar result was obtained for the posttest data. We created EF and IC composite scores at pretest (*M*s = 0.69 and 0.71, respectively) and posttest (*M*s = 0.75 and 0.79, respectively) assessments by taking the mean accuracy for all relevant tasks. Ceiling effects were not evident for the EF composite at pre- or posttest. In contrast, ceiling effects were evident for 7 and 12% of children for the IC composite at pre- and posttest assessments, respectively.

**TABLE 4:** Correlations among individual executive function tasks at pretest and posttest.

	1	2	3	4	5
1. ARR	−	0.29***	0.15*	0.15*	0.10^+^
2. SSS	0.22***	−	0.26***	0.16**	0.20***
3. AGNG	0.19***	0.26***	−	0.29***	0.16**
4. STS	0.12*	0.17***	0.22**	−	0.15**
5. PTP	0.24***	0.26***	0.24**	0.29**	−

^+^*p* < 0.10, *p < 0.05, **p < 0.01, and ***p < 0.001. Values below and above the diagonal are from the pretest (pairwise *N*s = 299–414) and posttest (pairwise *N*s = 271–384) assessments, respectively; ARR, arrows (inhibitory control); SSS, silly sounds stroop (inhibitory control); AGNG, animal go/no-go (inhibitory control); STS, something’s the same (attention shifting); PTP, pick the picture (working memory).

### Hierarchical Data Structure and Tests of Treatment Effects

A series of two-level (children nested in classrooms) unconditional multilevel models were estimated for each outcome at the posttest assessment (three-level models that considered an additional level of nesting of classrooms in centers were not supported by the data). Intraclass correlations (ICCs) were larger for EF and IC composites (0.14 and 0.13, respectively) than for individual tasks (ICCs ranging from 0.01 to 0.10). We retained the two-level parameterization for the evaluation of group differences.

A series of two-level conditional multilevel models were estimated for each outcome. As summarized in Table 5, children in the two treatment conditions did not differ at baseline (unadjusted comparisons) or at posttest (covariate adjusted comparisons). Individual EF tasks and the associated composite scores were similar between treatment conditions before and after treatment. Each of the two-level conditional multilevel models was reestimated four times. Language of assessment, school type (APBET, public), classroom type [PP1 (younger), PP2 (older)], and pretest ability were considered as moderators. Across these 28 additional models (7 outcomes × 4 moderators), there was no evidence for moderated treatment effects.

**TABLE 5:** Comparison of treatment conditions at pretest and posttest.

	Pretest				Posttest			
	RLPL *M*	Wait-list *M*	Comparison		RLPL LSM	Wait-list LSM	Comparison	
			*p* value	Cohen’s *d*			*p* value	Cohen’s *d*
EF composite	0.69	0.69	0.96	0.00	0.74	0.75	0.53	-0.07
IC composite	0.71	0.71	0.94	0.00	0.78	0.81	0.29	-0.13
Arrows	0.59	0.56	0.46	0.11	0.64	0.69	0.26	-0.13
Silly sounds stroop	0.81	0.82	0.80	-0.05	0.86	0.89	0.24	-0.14
Animal go/no-go	0.73	0.74	0.68	-0.03	0.88	0.87	0.71	0.03
Something’s the Same	0.64	0.65	0.48	-0.07	0.68	0.68	0.87	0.00
Pick the picture	0.69	0.67	0.30	0.17	0.69	0.70	0.27	-0.08

*RLPL, red-light purple-light treatment condition; Wait-list, comparison condition that implemented business as usual practices during the active study period; M, mean; LSM, least square mean (adjusted for pretest and simple reaction time); EF, executive function; IC, inhibitory control. Comparisons are based on the result of a 2-level hierarchical linear model in which students are nested in classrooms; Cohen’s *ds* effect sizes were computed by taking the difference in (least squares) means (treatment–wait-list) divided by the corresponding total sample standard deviation from the pretest assessment.*

## Discussion

To the best of our knowledge, this is the first study that explicitly targeted improvements in EF skills in early childhood in a LMIC context. In contrast to five previous studies that were conducted in high-income countries, children who were exposed to the RLPL intervention in Kenya did not demonstrate improvements in EF skills relative to children in the wait-list control condition. We included twice as many students as any previous evaluation, which helps to rule out low statistical power as an explanation for our failure to replicate results. We consider three possible explanations for our findings.

The first explanation for the lack of RLPL effects concerns the adaptation process. The RLPL training and materials that were used in this study were adapted for use in Kenya (see Supplementary Material). The team responsible for adapting the RLPL and delivering teacher training completed a 3-h training with the developers, who were aware of our plans to adapt materials. The primary adaptations focused on streamlining background information in the training manual and revising training and implementation materials into a format that was familiar to Kenyan preprimary teachers. Although teacher fidelity of implementation was high (see Tables 1, 2), we cannot rule out the adaptation of intervention materials, including reductions in the length of the training manual, as an explanation for null effects.

The second explanation for the lack of RLPL effects concerns measurement. Most previous evaluations of the RLPL program used the HTKS task as the primary outcome. The HTKS is positively correlated with performance-based measures of IC, working memory, and attention shifting, with *r*s ≈ 0.4–0.5 (McClelland et al., 2014). However, unlike traditional EF tasks, the HTKS involves a motor component. Two previous evaluations included a traditional EF assessment (i.e., a card sorting task). Schmitt et al. (2015) reported a smaller treatment effect for the Card Sorting task compared to HTKS, while Keown et al. (2020) reported the converse. The emphasis on motor activities in the RLPL may help to explain the consistent effects on the HTKS, which involves more motor inhibition than traditional cognitive EF assessments that we used here.

Another measurement-related explanation for our findings may be that our IC tasks were too easy for many children. The rates of ceiling effects that were observed in this sample were appreciably larger than in our previous studies in Kenya. Notably, the current study was conducted late in the Kenyan school year, which resulted in participants being relatively old for grade. Moreover, whereas our previous measurement-related work was conducted in four diverse counties in Kenya (Willoughby et al., 2019b), this study was conducted exclusively in Nairobi County, in schools with relatively higher socioeconomic status than in our previous work. The older age and socioeconomic advantage likely contributed to the strong performance on IC tasks. The ceiling effects were specific to individual IC tasks and do not fully explain the lack of findings for the IC composite (for which ceiling effects were less common), the overall EF composite, or the individual working memory and attention shifting tasks.

The third possible explanation for the lack of RLPL effects concerns the specific characteristics of preprimary classrooms in Kenya. Previous evaluations of the RLPL intervention were conducted in the United States or New Zealand, where the ratio of children to teachers is much smaller (e.g., 8–9:1) for 3- to 5-year-old children than was the case in this study (see Table 2). While the highly variable classroom sizes reflect the diversity of early learning environments in Kenya, large class sizes may have limited teachers’ ability to monitor individual children’s comprehension of and engagement in RLPL activities. Indeed, the item “teacher checked for student understanding” was the least frequently endorsed indicator of fidelity. Our study highlights the importance of attending to classroom size when considering classroom-based EF interventions for use with young children in Kenya.

Another possibility is that there were other classroom activities that were common to all classrooms that fostered children’s EF skill development. Although the Kenyan preprimary curriculum does not prioritize (or mention) EF as an explicit target, there is a strong emphasis on preacademic skill development, and all participating classes had previously participated in the Tayari intervention. We cannot rule out the possibility that ongoing supports for academic learning contributed to all children’s strong performance on EF tasks. Finally, the intervention may have been perceived as burdensome by some teachers. The RLPL activities were delivered during the outdoor activity period in the Kenyan academic schedule, which is typically a free period. Asking teachers to deliver RLPL activities during this period may have been perceived by some as additional work and may have contributed to lower implementation.

In sum, we documented an initial effort to deliver an EF-skills–focused program for use with preprimary-age children in Kenya. In contrast to five studies that were conducted in the United States and New Zealand, children who were exposed to the RLPL intervention did not demonstrate improvements in EF skills. Nonetheless, teachers were generally able to deliver RLPL games with high fidelity. We suspect that measurement and contextual issues likely explain the null results. Although we established the feasibility of delivering an EF-skills–focused intervention in preprimary classrooms in Kenya, our findings highlight important issues related to transporting efficacious interventions from high-income into LMIC contexts. We hope that the lessons described here will spur more work in this area, including the development of new EF interventions that are designed specifically for contexts like Kenya.

## Data Availability Statement

The datasets presented in this article are not readily available because requests for access to study data will be contingent on inputs from relevant institutional review boards. Requests to access the datasets should be directed to MW, mwilloughby@rti.org.

## Ethics Statement

The studies involving human participants were reviewed and approved by the National Commission for Science, Technology and Innovation and by the Kenya Medical Research Institute. Written informed consent to participate in this study was provided by the participants’ legal guardian/next of kin.

## Author Contributions

MW contributed to study design, supervised statistical analysis, and took primary responsibility for manuscript writing. BP contributed to study design, preschool sampling, supervision of in-country staffing, and manuscript writing. KK contributed to study design and manuscript writing. TN adapted intervention materials, trained teachers, conducted fidelity observations, oversaw data collection, and contributed to manuscript writing. CH adapted intervention materials and contributed to manuscript writing. SZ contributed to statistical analysis and manuscript writing. All authors contributed to the article and approved the submitted version.

## Conflict of Interest

The authors declare that the research was conducted in the absence of any commercial or financial relationships that could be construed as a potential conflict of interest.
