# Supplementary material for: Testing the Efficacy of the Red-Light Purple-Light Games in Preprimary Classrooms in Kenya
Source: Front Psychol. 2021 Mar 11;12:633049. doi: 10.3389/fpsyg.2021.633049 (PMC7990763; doi:10.3389/fpsyg.2021.633049)
Supplement: Supplementary file 1 [file Data_Sheet_1.PDF]

Supplementary Materials – Intervention Materials Adaptation and Pilot Testing

The intervention team determined that all the RLPL games were appropriate for the Kenyan context. Content adaptations were minimal and included things like replacing songs with ones that were familiar to Kenyan children. The intervention team also replaced some specific language to better fit the Kenyan context (e.g., "conductor of an orchestra" became "leader of a band"; "baton" became "stick"). The teacher guides and student materials that were obtained from the RLPL developers were adapted for the Kenyan context. The adaptation was guided by previous work in Kenya, which emphasized the value of streamlining the scripting of lesson plans (Piper et al, 2018). The original RLPL teacher booklet, which was 80 pages and included both overall guidance and sample scripts for each session, was reduced to 44 pages and aligned to the level of scripting that was familiar to teachers based on their participation in the Tayari program (a copy of the revised teacher training materials are available upon request from the first author). For example, the RLPL manual provides both sample scripts and session guides for teachers. We elected to only provide scripted guidance to teachers in a familiar format. The organization of and frequency with which games were introduced and delivered remained the same. Adaptations were made to the list of materials required, as it is common for Kenyan classrooms to have reduced access to a large variety of materials. For example, since most teachers do not have music players in the classroom (e.g., CD player), the team encouraged teachers to sing songs rather than rely on a device to play songs. Additionally, the team provided updated graphics (e.g., of animals) to make them more recognizable to Kenyan children and replaced some of the welcome and introduction songs with songs that Kenyan children know. All participating teachers received a core package of materials that included the printable material and colored paper required for some of the games so that teachers did not have to procure or create materials on their own.

After the first RLPL materials adaptation process, the materials were piloted in 5 schools. The feedback from teachers and learners informed further content and materials adaptations, which primarily included streamlining or simplifying the scripts. Teachers also suggested key formatting decisions that would increase the size of some graphics given the need to display them to the whole class given the larger sample sizes. Additional revisions were made based on the large number of students and limited space (e.g., "*have learners lie on their mats*" was replaced with "*have learners place their heads on their desks"*).

Word Count: 428
